# Molecular advances in bud dormancy in trees

**DOI:** 10.1093/jxb/erae183

**Published:** 2024-06-03

**Authors:** Jihua Ding, Kejing Wang, Shashank Pandey, Mariano Perales, Isabel Allona, Md Rezaul Islam Khan, Victor B Busov, Rishikesh P Bhalerao

**Affiliations:** National Key Laboratory for Germplasm Innovation & Utilization of Horticultural Crops, Hubei Hongshan Laboratory, Hubei Engineering Technology Research Center for Forestry Information, College of Horticulture and Forestry, Huazhong Agricultural University, 430070, Wuhan, China; National Key Laboratory for Germplasm Innovation & Utilization of Horticultural Crops, Hubei Hongshan Laboratory, Hubei Engineering Technology Research Center for Forestry Information, College of Horticulture and Forestry, Huazhong Agricultural University, 430070, Wuhan, China; Umeå Plant Science Centre, Department of Forest Genetics and Plant Physiology, Swedish University of Agricultural Sciences, 901 83 Umeå, Sweden; Centro de Biotecnología y Genómica de Plantas, Universidad Politécnica de Madrid, Centro Nacional Instituto de Investigación y Tecnología Agraria y Alimentaria, CNINIA (CSIC), Madrid, Spain; Departamento de Biotecnología-Biología Vegetal, Escuela Técnica Superior de Ingeniería Agronómica, Alimentaria y de Biosistemas, Universidad Politécnica de Madrid, Madrid, Spain; Centro de Biotecnología y Genómica de Plantas, Universidad Politécnica de Madrid, Centro Nacional Instituto de Investigación y Tecnología Agraria y Alimentaria, CNINIA (CSIC), Madrid, Spain; Departamento de Biotecnología-Biología Vegetal, Escuela Técnica Superior de Ingeniería Agronómica, Alimentaria y de Biosistemas, Universidad Politécnica de Madrid, Madrid, Spain; College of Forest Resources and Environmental Science, Michigan Technological University, Houghton, MI, USA; College of Forest Resources and Environmental Science, Michigan Technological University, Houghton, MI, USA; Umeå Plant Science Centre, Department of Forest Genetics and Plant Physiology, Swedish University of Agricultural Sciences, 901 83 Umeå, Sweden; University of Western Australia, Australia

**Keywords:** Abscisic acid, bud break, *DAM/SVL*, dormancy, *FT*, gibberellins, plasmodesmata

## Abstract

Seasonal bud dormancy in perennial woody plants is a crucial and intricate process that is vital for the survival and development of plants. Over the past few decades, significant advancements have been made in understanding many features of bud dormancy, particularly in model species, where certain molecular mechanisms underlying this process have been elucidated. We provide an overview of recent molecular progress in understanding bud dormancy in trees, with a specific emphasis on the integration of common signaling and molecular mechanisms identified across different tree species. Additionally, we address some challenges that have emerged from our current understanding of bud dormancy and offer insights for future studies.

## Teaser

We present an overview of recent molecular advances in bud dormancy in trees, with a particular focus on the integration of common molecular mechanisms identified among different tree species.

## Introduction

The annual dormancy in perennial woody plants is a widespread adaptative strategy that allows plants to survive in seasonally unfavorable conditions (Cooke *et al.*, 2012). It is therefore a crucial agronomic trait that significantly impacts the performance of many horticultural crops and the health and productivity of forest ecosystems (Yang *et al.*, 2021). Various definitions of dormancy have been proposed, but basically dormancy involves suppression of growth by internal and/or external factors (Rohde and Bhalerao, 2007). One well-known classification of dormancy was proposed by Lang *et al.* (1987), who categorized it into three types: para-dormancy, endo-dormancy, and eco-dormancy, based on the factors that induce dormancy. The analysis of dormancy becomes increasingly complex and diverse as we delve deeper into the subject. In polycarpic plants, where multiple growth and developmental axes co-exist due to the activity of different meristems, the seasonal dormancy of trees manifests itself in vegetative buds, flower buds, mixed buds, fruits, vascular cambium, etc. Each of these bud types undergoes its unique dormancy process in terms of factors regulating it, although overlaps in regulatory mechanisms are observed (Savage and Chuine, 2021). For instance, it has been known for a long time that there are differences in the intensity of dormancy between flower buds and vegetative buds in many fruit trees (Hatch and Walker, 1969; Walker, 1970). In recent years, an increasing number of studies have supported the notion that in *Rosaceae* fruit trees, the dormancy of the vegetative bud and flower bud is not synchronized (Fadon *et al.*, 2018; Rothkegel *et al.*, 2020; Canton *et al.*, 2022). While floral development progresses in the flower buds, in the apical vegetative bud there is typically no growth. Nonetheless, chilling remains crucial for floral bud development, as floral organs are unable to develop normally without exposure to chilling temperatures (Atkinson *et al.*, 2013; Kumar *et al.*, 2017). These findings indicate that the traditional definition of dormancy (e.g. no visible signs of growth) may not fully apply to flower buds at least of *Rosaceae* trees. Additionally, the bud phenology and cambium phenology progress through different phases. In conifers, it has been observed that bud break can occur either before or after the onset of cambium activity (Rensing and Owens, 1994; Rossi *et al.*, 2009), while in beech (*Fagus sylvatica*) trees, cambial reactivation occurs immediately after bud break (Cufar *et al.*, 2008). These observations indicate that tree dormancy may represent a systemic adaptation involving unique and coordinated mechanisms across different organs (Savage and Chuine, 2021).

**Fig. 1.**
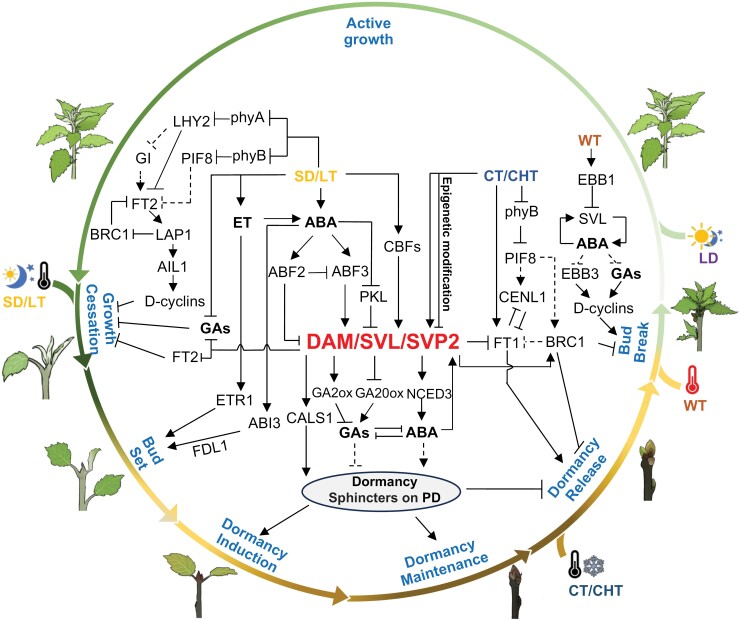
Molecular networks of the bud dormancy process (most are based on studies from *Populus*). The illustrations outside the circle display the representative shoot and bud morphologies at different stages of the dormancy cycle. The corresponding stages are indicated inside the circle with blue characters. During late spring and summer, long days (LD) promote vegetative growth (active growth). In response to short days (SD) or low temperatures (LT), the tree will cease growth (growth cessation), and the shoot apices develop into buds (bud set). Prolonged SD and/or LT will induce bud dormancy (dormancy induction). The dormancy state will be maintained (dormancy maintenance) until cold signals (chilling temperatures, CHT) arrive in winter, triggering bud dormancy release (dormancy release). The bud is poised to break when exposed to warm temperature (WT) in spring (bud break). LD and WT further promote growth, repeating the cycle. The model for *DAM/SVL/SVP2*-centered molecular regulation networks of the bud dormancy cycle is depicted inside the circle. The LD signal is sensed by photoreceptors (phyA and phyB) that, along with genes such as *PtLHY*, *PtGI*, and *PHYTOCHROME INTERACTING FACTOR 8* (*PtPIF8*), measure the day length. This LD signal induces *FT2* expression in leaves and increases the levels of the growth hormones GAs. The FT2 protein can travel from the leaf through the phloem to the shoot apex, where it can interact with FDL1 to induce the expression of *LAP1* and *AIL1*, activating cell cycle genes (D-cyclins), and maintain active growth in the shoot apex. GAs can also promote growth both through and independently of *FT2*. SDs suppress this pathway, leading to growth cessation. SDs also triggers ethylene and ABA signaling, promoting bud development and maturation through ETR1 and FDL1. Additionally, ABA induces bud dormancy via the genes *ABF*, *PKL*, and *DAM/SVL/SVP2*, which inhibit growth-promoting GA signaling, increase ABA levels through activating the ABA synthesis gene *NCED3*, and activate PD blockage-related genes such as *CALS*. Both SDs and LDs also can induce *CBF* genes, which can directly promote the expression of *DAM/SVL/SVP2*. Epigenetic modification may play a role in the dormancy release process by partially repressing *DAM/SVL/SVP2* levels during chilling exposure. Chilling also induces *FT1* expression, both through and independently of *DAM/SVL/SVP2*. *PHYB* signal acts as a thermosensor, helping active *FT1* during dormancy release. Warm spring temperatures induce *EBB1*, inhibiting the SVL–ABA loop, leading to *EBB3* activation and GA biosynthesis, ultimately triggering the induction of cell cycle genes and bud break. Arrowheads represent positive effects, blocked arrows denote negative effects, and dashed lines represent indirect regulation or uncertain pathways.

Our current knowledge of the molecular mechanisms involved in the bud dormancy process is primarily derived from studies of vegetative buds, and has been categorized into a series of progressive events (Fig. 1): following growth cessation at the apex (growth cessation) and formation of buds (bud set), dormancy is established in buds (dormancy induction). Dormancy is then maintained (dormancy maintenance) and gradually released (dormancy release) when competence to grow is restored, followed by reinitiation of growth (bud break) (Ding and Nilsson, 2016; Goeckeritz and Hollender, 2021; Singh *et al.*, 2021). Over the past few decades, significant advancements have been made in understanding the molecular mechanisms underlying the bud dormancy process in various plant species. In this review, we provide a summary of these recent molecular advances in bud dormancy (particularly apical bud dormancy) in trees, with a specific emphasis on the integration of common signaling and molecular mechanisms identified among different tree species.

## External environmental and endogenous signals driving the bud dormancy cycle

Annual bud dormancy is a consequence of the interplay between environment signals and endogenous mediators of these external factors. Photoperiod and temperature act as the primary triggers for this phenomenon, at least in trees from boreal and temperate regions. The influences of these two environmental signals on the bud dormancy cycle are complex. Moreover, the environmental requirements for the same process can differ among species (Ding and Nilsson, 2016; Triozzi *et al.*, 2018; Singh *et al.*, 2021). For example, in poplar (*Populus* sp.) and spruce (*Picea* sp.) trees, a short-day photoperiod alone is sufficient to trigger both growth cessation and dormancy (Heide, 1974; Espinosa-Ruiz *et al.*, 2004), while low temperature appears to play a significant role in these two processes in many *Rosaceae* fruit trees (Heide and Prestrud, 2005; Yang *et al.*, 2021). In certain deciduous trees, it has been noted that short days in conjunction with high temperatures can intensify bud dormancy depth and postpone bud break in spring compared with low temperatures, indicating an interaction between photoperiod and temperature in regulating bud dormancy in these plants (Westergaard and Eriksen, 1997; Heide, 2003; Kalcsits *et al.*, 2009; Malyshev, 2020). Once established, chilling is typically necessary for dormancy release, but the specific chilling requirement varies across species and even within populations of the same species. Moreover, it has been observed that the chilling requirement for dormancy release is determined not solely by the duration of chilling, but also by the temperature conditions before, during, and after the chilling period (North *et al.*, 2022). Studies in birch (*Betula* sp.) have demonstrated that the minimum temperature required for bud break decreases with prolonged chilling duration (Junttila *et al.*, 2003; Junttila and Hanninen, 2012). This may serve as an adaptative strategy for northern trees to facilitate spring bud break even under low temperature conditions. Once dormancy is released, growth competence is restored, with relatively warm temperatures being instrumental in driving bud break and blooming in spring. Numerous studies have identified a negative correlation between chilling accumulation during dormancy release and heat requirement for bud break. Extended exposure to chilling leads to a reduction in the time taken for bud break under similar growth-promoting conditions (Heide, 1993; Junttila *et al.*, 2003; Okie and Blackburn, 2011; Nanninga *et al.*, 2017). Additionally, warmer temperature accelerates bud break, but the requirement for specific high temperature is not absolute. Prolonged chilling can lead to bud break occurring at the chilling temperature itself in birch (Heide, 1993; Myking and Heide, 1995). Therefore, in addition to temperature and photoperiod, time also serves as an important factor for various developmental stages within the dormancy cycle.

In addition to external environmental signals, various endogenous factors including phytohormones, sugars, and oxidative stresses also play roles in different stages of the dormancy cycle, potentially acting as mediators of external cues. The plant hormones abscisic acid (ABA) and gibberellins (GAs) are the best studied endogenous regulators of bud dormancy. ABA and GA antagonistically regulate bud dormancy induction, maintenance, and release (Yue *et al.*, 2018; Liu and Sherif, 2019; Veerabagu *et al.*, 2023). It is commonly observed that endogenous ABA levels (and possibly ABA response) rise during dormancy establishment and decline towards dormancy release (Ruttink *et al.*, 2007; Karlberg *et al.*, 2010; Li *et al.*, 2018; Zheng *et al.*, 2018a). Conversely, the dynamics of GA contents are often inversely correlated with ABA in many woody species, decreasing at dormancy induction and increasing again during dormancy release (Cooke *et al.*, 2012; Zhuang *et al.*, 2013; Wen *et al.*, 2016; Zheng *et al.*, 2018b). Application of exogenous ABA in several tree species can promote dormancy induction and delay bud break (Mielke and Dennis, 1978; Li *et al.*, 2018). In contrast, exogenous GAs (such as GA_3_ and GA_4_) accelerate bud dormancy release and facilitate bud break (Rinne *et al.*, 2011; Zhuang *et al.*, 2013; Zheng *et al.*, 2018b; Zhang *et al.*, 2021). Direct evidence of ABA’s role in regulating bud dormancy has been demonstrated in studies of hybrid aspen trees, with reduced ABA responses due to the expression of the dominant-negative *abi1-1* allele of *ABSCISIC ACID-INSENSITIVE1* (*ABI1*), a key ABA signaling gene, resulting in the failure to establish dormancy (Tylewicz *et al.*, 2018). Similarly, overexpression of *GA2-oxidase* (*GA2ox*), the key enzyme for GA metabolism, in hybrid poplar (*Populus tremula×Populus alba*) leads to accelerated bud set and delayed bud flush (Zawaski *et al.*, 2011; Singh *et al.*, 2018). Furthermore, ABA induces dormancy partially through GA metabolic pathways, as demonstrated by the restoration of dormancy in *abi1-1* plants upon overexpression of *GA2ox* (Singh *et al.*, 2019), indicating the interplay between ABA and GA in the dormancy process. In addition to the extensive studies on the antagonistic functions of ABA/GA in dormancy induction and release, these two phytohormones also play important roles in other stages of the dormancy cycle. For instance, GAs have been identified as critical factors in short-day (SD)-induced growth cessation, functioning both in conjunction with and independently of *FLOWERING LOCUS T2* (*FT2*) (Eriksson *et al.*, 2000, 2015; Misckolzi *et al.*, 2019; Gomez-Soto *et al.*, 2021a; Andre *et al.*, 2022b). Another significant gene involved in ABA signaling, *ABSCISIC ACID-INSENSITIVE3* (*ABI3*), has been found to play a crucial role in bud formation (Rohde *et al.*, 2002). ABI3 works in conjunction with FD-like 1 (FDL1) to promote the adaptive response and bud maturation (Tylewicz *et al.*, 2015).

Furthermore, other plant phytohormones such as ethylene, auxin (indole-3-acetic acid, IAA), cytokinin (CK), and jasmonates (JAs) have also been found to participate in the dormancy process (Liu and Sherif, 2019). For example, the expression of a dominant-negative version of *ETHYLENE TRIPLE RESPONSE 1* (*ETR1*) in birch prevents the closure of apical buds, indicating the involvement of ethylene in bud formation (Ruonala *et al.*, 2006). IAA and CK, two growth promoter hormones, have been reported to increase during dormancy release in many tree species (Cutting *et al.*, 1991; Nagar and Sood, 2006; Baba *et al.*, 2011; Qiu *et al.*, 2013; Zhang *et al.*, 2018). In particular, they are known to play essential roles in regulating cambium stem cell dormancy dynamics (Baba *et al.*, 2011; Immanen *et al.*, 2016; Bhalerao and Fischer, 2017). Involvement in axillary bud dormancy regulation in annual plants suggests that auxin and CK may have crucial roles in activating the cell cycle machinery for activation of axillary bud outgrowth, although similar roles in apical buds have not been functionally demonstrated (Kotov *et al.*, 2021). While both JA and ABA are known to synergistically participate in certain stress response processes, JA probably acts antagonistically to ABA in the regulation of dormancy release. Application of methyl-jasmonate (MeJA) has been shown to promote wheat grain germination by inhibiting the expression of the ABA biosynthesis gene *9-cis-epoxycarotenoid dioxygenase 1* (*TaNCED1*) (Jacobsen *et al.*, 2013). Likewise, levels of JA gradually increase during bud dormancy release in beech trees (Juvany *et al.*, 2015). Recent studies in pear (*Pyrus pyrifolia*) confirmed that JA, along with another plant hormone, brassinosteroid (BR), positively contribute to pear bud dormancy release (Wang *et al.*, 2024). In summary, these advancements suggests that plant hormones interact intensively with each other and act coordinately in the regulation of bud dormancy. However, it is important to note that most of these hormone-related studies have primarily remained at the physiological level (Liu and Sherif, 2019). As numerous other physiological processes overlap temporally with dormancy (induction and release), more genetic evidence is needed to unravel the endogenous role of hormones (especially of hormones other than ABA and GA) and integration into molecular pathways regulating bud dormancy.

In addition to plant hormones, sugar (sucrose) has been proposed to act as a trigger for axillary bud outgrowth in many annual plants (referred to as para-dormancy in perennial plants) (Lang *et al.*, 1987; Barbier *et al.*, 2015, 2019). Also, the dynamic changes in sugar levels correlate well with potato tuber bud dormancy release (referred to as endo-dormancy), and modification of accumulation or signaling of sugars in transgenic tubers can alter the duration of dormancy (Danieli *et al.*, 2023). Trehalose 6-phosphate (Tre6P) has been identified as a mediator of sugar signal that links growth and development to carbon status (Fichtner *et al.*, 2017, 2021). Tre6P may regulate dormancy by inhibiting the sucrose non-fermenting kinase 1 (SnRK1) complex, a central integrator that coordinates energy balance, metabolism, and growth (Baena-Gonzalez *et al.*, 2007). The ability of perennial woody plants to resume growth in the spring season is also reliant on non-structural carbohydrates (NSCs; i.e. sugars and starch) (Tixier *et al.*, 2019). Studies in many trees have shown that the dynamic changes of carbohydrate metabolism align well with bud dormancy release (Tixier *et al.*, 2018). While the influence of NSC on the bud dormancy release process in perennial trees is not fully understood, insights from studies in annual plants can provide some clues. For example, sugar accumulation induced by chilling was also associated with plasmodesmata (PDs) closure in the potato tuber dormant bud meristem (Danieli *et al.*, 2023), although whether the effect of sugar accumulation on PDs was direct is not known. Interestingly, the target of rapamycin (TOR), another key protein kinases that acts antagonistically with SnRK1 in plant growth and nutrient status interactions (Xiong *et al.*, 2013), was also found to influence plasmodesmatal aperture and source-to-sink transport (Brunkard *et al.*, 2020). Since the orchestrated movement via PDs is essential for bud dormancy establishment and release (see next section), these observations, taken together, may suggest that changes in sugar levels could be involved in bud dormancy release via their effects on cell-to-cell symplastic connections. Furthermore, carbohydrate metabolism is linked to reactive oxygen species (ROS) accumulation, which has also been implicated in dormancy release (Beauvieux *et al.*, 2018). In summary, dormancy is a complex process with highly correlated interactions among hormones, carbohydrate metabolism, ROS, and cell wall properties in the bud dormancy process. Future studies are, however, necessary to address the molecular mechanisms underlying the role of these signals in dormancy regulation.

## Plasmodesmata and their role in dormancy regulation

Plant growth and development rely on both local cues and signals acting locally or systemically. PDs are intercellular channels lined with plant membrane that facilitate cell-to-cell communications, enabling the transport of molecular signals to coordinate developmental and environmental responses (Sager and Lee, 2014). PDs can transition between open and closed configurations, regulating the movement of mobile regulatory factors and molecules between cells (Bayer and Benitez-Alfonso, 2024). During tree dormancy, PDs are blocked in buds as well as in vascular tissue such as sieve plates in phloem (Evert, 1990; Savage and Chuine, 2021). The conductivity of PDs is partially, if not entirely, dependent on the callose (1,3-β-d-glucan) accumulation in the cell wall surrounding the PDs (Li *et al.*, 2021). The levels of callose are controlled by two counteracting enzymatic activities, callose synthase and hydrolases. Phytohormones have been found to play important roles in plasmodesmata-mediated trafficking through deposition or removal of callose (Sankoh and Burch-Smith, 2021). This PD–hormone interplay has been implicated in bud dormancy regulation (Rinne *et al.*, 2001, 2011; Sankoh and Burch-Smith, 2021). Studies in hybrid aspen have demonstrated that SD-induced ABA accumulation contributes to the callose deposition on PDs by promoting the expression of the *callose synthase 1* (*PtCALS1*) gene, leading to PD closure and dormancy onset (Tylewicz *et al.*, 2018; Singh *et al.*, 2019). A similar mechanism has been reported in growth transitions (from slow to fast growth) of lily bulbs. Prolonged cold treatment of lily buds leads to a reduction in callose levels, resulting in the opening of PDs to facilitate bud sprouting (Pan *et al.*, 2023). In potatoes, the maintenance of axillary bud dormancy in aerial nodes is regulated by callose deposition around the PDs. This deposition blocks tuber-inducing activity in aerial nodes, preventing buds from competing in sink strength with stolons (Nicolas *et al.*, 2022). Studies in hybrid aspen and lily have demonstrated a role for epigenetic regulation in PD dynamics. In lilies, histone acetylation (H3K9ac) maintains high expression of *LoCALS3* in dormant buds. Long-term cold treatment induces an increase in H3K27 trimethylation (H3K27me3) in the *LoCALS3* locus, leading to reduced *LoCALS3* expression. The deposition of H3K27me3 is mediated by the Polycomb repressive complex 2 (PRC2), recruited by the transcription factor (TF) NUCLEAR FACTOR Y, SUBUNIT A7 (LoNFYA7), which binds to the *LoCALS3* promoter (Pan *et al.*, 2023). In hybrid aspen, ABA can induce the expression of *PtCALS1* and suppress glucanases that break down callose, leading to the blockage of PDs (Tylewicz *et al.*, 2018). Down-regulation of *PICKLE* (*PKL*), a chromatin remodeler that facilitates epigenetic marks and represses gene expression, restores PD closure and bud dormancy defects in *abi1-1* plants. This suggests that ABA mediates PD closure and bud dormancy by suppressing *PKL*. These findings suggest a common mechanism of the PD intercellular trafficking mechanism related to epigenetic modifications in bud dormancy control. Moreover, the ectopic expression of PD-located protein 1 (PDLP1), a receptor-like membrane protein targeted to PDs, can also restore the PD closure and dormancy in the *abi1-1* mutant of hybrid aspen (Tylewicz *et al.*, 2018). This suggests involvement of multiple molecular layers of PD intercellular trafficking in dormancy regulation (Li *et al.*, 2021). Recent studies in *Eucalyptus dunnii*, a subtropical tree that does not undergo dormancy, have shown that under cold winter conditions (growth arrest, corresponding to eco-dormancy), subtle callosic sphincters were observed at the PDs (Dai *et al.*, 2023), further supporting the link between callose deposition at PDs and dormancy. In contrast, regulation of PD opening in trees is not clear. While application of exogenous GAs to axillary buds does reduce sphincters during dormancy (Rinne *et al.*, 2011), the *in vivo* role of GAs in the shoot apex and the molecular players mediating this response remain largely unknown. It is also unclear whether this process involves a similar epigenetic mechanism to that observed in shoot apical meristems in lily buds. However, functional evidence is needed to elucidate whether GA is indeed the endogenous mediator of low temperature-induced PD opening, the specific mechanisms by which GA influences PD dynamics in bud dormancy regulation, and whether epigenetic modifications play a role in this process.

## *DAM/SVP* genes and related transcription factors associated with bud dormancy

In the past decade, extensive research has focused on identifying genetic regulators that control bud dormancy. Among these regulators, a group of TF genes, known as the *DORMANCY-ASSOCIATED MADS-box* (*DAM*) in *Rosaceae* fruit trees, *SHORT VEGETATIVE PHASE-like* (*SVL*) in *Populus*, and *SHORT VEGETATIVE PHASE 2* (*SVP2*) in kiwifruit (*Actinidia* sp.), have been widely studied across different taxa (Wu *et al.*, 2017b; Falavigna *et al.*, 2019; Singh *et al.*, 2019). These *DAM/SVP* genes are closely related to *SHORT VEGETATIVE PHASE* (*SVP*) and *AGAMOUS*-*LIKE 24* (*AGL24*), two MADS box genes implicated in floral transition and development in Arabidopsis (Hartmann *et al.*, 2000; Yu *et al.*, 2002*). Populus SVL* has been intensively studied and shown to play multifaceted roles in different dormancy phases, primarily acting as a promoter for dormancy induction and maintenance (Singh *et al.*, 2018, 2019; Busov, 2019; Andre *et al.*, 2022b). The *DAM* genes were initially discovered in an *evergrowing* (*evg*) mutant peach, which is insensitive to environmental signals and can grow continuously without undergoing growth cessation (Rodriguez *et al.*, 1994; Bielenberg *et al.*, 2008). Genetic studies in *Rosaceae* fruit trees, such as peach, apple (*Malus×domestica*), pear (*Pyrus communis*), Japanese apricot (Prunus mume), apricot (*Prunus armeniaca*), and sweet cherry (*Prunus avium*), have revealed the presence of quantitative trait loci (QTLs) that coincide with location of *DAM* genes, indicating their involvement in dormancy regulation (Castede *et al.*, 2015; Allard *et al.*, 2016; Gabay *et al.*, 2017; Kitamura *et al.*, 2018; Falavigna *et al.*, 2019; Cantin *et al.*, 2020). A recent genome-wide association study (GWAS) analysis in peach confirmed that *PpDAM6* overlaps with the *EVG* locus and functions similarly to *SVL* in hybrid aspen. *PpDAM6* promotes dormancy, and compromising its function results in a reduced amount of chilling exposure for dormancy release (Zhao *et al.*, 2023). However, unlike *SVL*, *PpDAM6* does not eliminate the chilling requirement entirely (Zhao *et al.*, 2023). Similarly, overexpression of both the *MdDAMb* from apple and *PmDAM6* from Japanese apricot can suppress bud break in apple (Wu *et al.*, 2017a; Yamane *et al.*, 2019). Conversely, transgenic apple plants with down-regulated *MdDAM1* and *MdDAM4* genes show no terminal bud formation and, consequently, dormancy induction (Moser *et al.*, 2020). In addition to *Rosaceae* fruit trees, *DAM/SVP* genes have also been identified in kiwifruit, and both physiological and genetic analysis have confirmed that kiwifruit *SVP2* can prevent premature bud break in axillary buds during dormancy (Wu *et al.*, 2012, 2017a). Consistent with their roles in dormancy regulation, *DAM/SVP* genes exhibit distinct seasonal expression pattens, with peak expression occurring during dormancy and being repressed by chilling temperatures (Falavigna *et al.*, 2019). These expression profiles are somewhat consistent even among different species. Collectively, these findings suggest that *DAM/SVP* genes have an important role in various aspects of dormancy and possibly bud break.

In addition to *DAM*/*SVP* genes, several other TF genes have also been identified that are involved in dormancy or bud break process. These include clade MADS-box TF genes such as *SUPPRESSOR OF OVEREXPRESSION OF CONSTANS 1* (*SOC1*)*-LIKE* and *FLOWERING LOCUS C* (*FLC*)*-LIKE* found in kiwifruit, apple, and poplar (Voogd *et al.*, 2015, 2022; Wu *et al.*, 2019; Gomez-Soto *et al.*, 2021b; Nishiyama *et al.*, 2021), as well as AP2/Ethylene responsive factors *EARLY BUD-BREAK 1* (*EBB1)* and *EARLY BUD-BREAK 3* (*EBB3*) from poplar (Yordanov *et al.*, 2014; Busov *et al.*, 2016; Azeez *et al.*, 2021). C-repeat binding factor genes (*CBF/DREB*) from pear and apple (Artlip *et al.*, 2019; Li *et al.*, 2019), bZIP-like TF genes (*FDL1*, *ABF*) from poplar and pear (Tylewicz *et al.*, 2015; Yang *et al.*, 2020, 2023; Sheng *et al.*, 2022), TCP genes (*TCP18/BRC1*) from poplar, blueberry, and potato, and a WRKY TF gene (*VvWRKY37*) from grape wine have also been identified as playing roles in dormancy regulation (Tylewicz *et al.*, 2018; Maurya *et al.*, 2020; Nicolas *et al.*, 2022; Wang *et al.*, 2022; Li *et al.*, 2023). These TF genes have distinct functions. *EBB1* and *EBB3* promote cell proliferation in the shoot apical meristem (SAM) and leaf primordia in poplar (Yordanov *et al.*, 2014; Azeez *et al.*, 2021). In Arabidopsis, *BRC1* is known to play a crucial role in axillary bud dormancy (Gonzalez-Grandio *et al.*, 2013) and its orthologs in perennial trees such as poplar and blueberry trees negatively regulate bud break (Tylewicz *et al.*, 2018; Maurya *et al.*, 2020; Li *et al.*, 2023). In some deciduous fruit trees such as apple and peach, low temperatures rather than short photoperiods are the primary environmental signals triggering growth cessation and dormancy (Heide and Prestrud, 2005), and thus it is interesting to note that *CBF* genes, which were initially identified as key regulators of cold response, can affect bud set and bud break when overexpressed. For instance, overexpression of peach *PpCBF2* in apple induces early bud set and delays bud break (Wisniewski *et al.*, 2015). Overexpression of *AtCBF1* in hybrid aspen results in dwarfing, suggesting reduced GA levels, which might explain delayed bud break (Benedict *et al.*, 2006). Further analysis using loss-of-function approaches is necessary to elucidate the direct effects of these TFs on bud set and bud break regulation.

The molecular pathways targeted by *DAM/SVP* genes have been partially elucidated. *Populus SVL*, for instance, has been found to mediate the SD-induced growth cessation by repressing *FT2* and GA biosynthesis pathways in the leaves (Andre *et al.*, 2022b), whereas it promotes bud dormancy by activating the ABA pathway in the shoot apex (Singh *et al.*, 2019), and represses bud break by antagonistically acting on ABA/GA signals (Singh *et al.*, 2018). Much like *SVL*, fruit *DAM* genes have been proposed to induce the expression of key enzymes for ABA biosynthesis and callose deposition (Yamane *et al.*, 2019; Zhao *et al.*, 2023). In kiwifruit, overexpression of *SVP2* also leads to transcriptome-level changes in the ABA and dehydration response pathways, with ChIP-sequencing (ChIP-seq) analysis demonstrating the direct binding of *SVP2* to genes related to ABA and drought response pathways (Wu *et al.*, 2017b, 2018). These findings suggest a conserved regulatory network involving ABA metabolism and symplasmic communication in dormancy buds, with *DAM/SVP* genes acting to promote dormancy. Conversely, *DAM/SVL* down-regulation by chilling could facilitate dormancy release (Fig. 1). However, whether down-regulation of *DAM/SVL* is sufficient and necessary for dormancy release/bud break remains to be evaluated; for example, *SVL* overexpression in hybrid poplar did not appreciably affect bud break under natural conditions (Goralogia *et al.*, 2021).

Several other TF genes have been reported to be directly or indirectly involved in *DAM*/*SVP* and ABA/GA signal networks (Fig. 1). For instance, poplar *EBB1* can directly suppress *SVL*, while *SVL* can up-regulate *BRC1*, which negatively regulates bud break by acting on GA and ABA pathways (Singh *et al.*, 2018; Azeez *et al.*, 2021). Additionally, *in vitro* assays in fruit trees have also shown that the ABA downstream signaling factors such as ABF2 and ABF3 can activate the expression of *DAM* genes during dormancy induction and release (Wang *et al.*, 2020; Yang *et al.*, 2020, 2021). Furthermore, the effect of *PpCBF2* overexpression on dormancy is associated with its effect on the expression of *MdDAM* and *MdEBB* genes in apple (Wisniewski *et al.*, 2015). Furthermore, *PmCBF* genes in *P. mume* have been found to bind to the promoter of *PmDAM6* and activate its expression directly, and thus co-regulate bud dormancy in response to cold temperatures (Zhao *et al.*, 2018). Overall, these studies suggest a shared regulatory network involving *DAM/SVP* genes and related TFs across various woody perennial species, although the understanding of this regulatory network is still evolving (Fig. 1). As outlined above, numerous studies have shown that *DAM/SVP* genes promote dormancy and their expression is down-regulated by chilling. Intriguingly though, factors (with the possible exception of *FT1*; see below) that are up-regulated by chilling and are also essential for dormancy release have not been reported. Thus, it remains to be seen whether such factors up-regulated by chilling also function in addition to *DAM/SVP* down-regulation in dormancy release.

## Dual roles of *PEBP* family genes in regulation of seasonal activity–dormancy growth and flowering

The annual vegetative activity–dormancy growth cycle in trees involves coordination with other development events, such as seasonal flowering. Genes from the phosphatidylethanolamine- binding protein (PEBP) family, which consists of three subfamilies in angiosperms [*FLOWERING LOCUS T* (*FT*), *TERMINAL FLOWER1* (*TFL1*)/*CENTRORADIALIS* (*CEN*)/*BROTHER OF FT AND TFL1* (*BFT*), and *MOTHER OF FT AND TFL1* (*MFT*)] (Bennett and Dixon, 2021), have been shown to function not only in flowering time and plant architecture regulation in diverse angiosperms (Lifschitz *et al.*, 2014; Zhu *et al.*, 2021), but also in vegetative phenology in trees (Pin and Nilsson, 2012; Ding and Nilsson, 2016; Nilsson, 2022; Wang and Ding, 2023). In *Populus*, studies have demonstrated the involvement of *PEBP* family members in various stages of seasonal active–dormancy growth transition. Two *FT* paralogs (*FT1* and *FT2*) exist in *Populus*, with each paralog having distinct functions. The functional differentiation between *FT1* and *FT2* is characterized by changes in their annual expression pattern and divergence in their known gene regulatory networks (Hsu *et al.*, 2011). *FT2* plays a key role in SD-induced growth cession in autumn (Bohlenius *et al.*, 2006). Over the past two decades, extensive research has been conducted on the genetic and molecular network of *FT2*-centered photoperiodic control of growth cessation in *Populus*. This network shows remarkable conservation with the photoperiodic control of flowering time in Arabidopsis (Ding and Nilsson, 2016; Singh *et al.*, 2021; Nilsson, 2022). This process involves the perception of light signals by the phytochromes (phyA and phyB) (Kozarewa *et al.*, 2010; Ding *et al.*, 2021), integration into internal circadian clock genes such as *LATE ELONGATED HYPOCOTYL* (*PtLHY*), *TIMING OF CAB EXPRESSION* (*PtTOC1*), and *GIGANTEA* (*PtGI*), and the measurement of the day length (Ding *et al.*, 2018; Ramos-Sanchez *et al.*, 2019; Alique *et al.*, 2024). The signals output from the circadian clock specifically induce the expression of *FT2* in leaves under long-day photoperiods. The FT2 protein can move from the leaf through the phloem to the shoot apex, where it can interact with FDL1 to induce the expression of *LIKE APETALA 1* (*LAP1*) and *AINTEGUMENTA-LIKE1* (*AIL1*) (Miskolczi *et al.*, 2019). This, in turn, activates cell cycle genes and maintains active growth in the shoot apex (Fig. 1). In contrast, the *FT1* gene plays a critical role in the reactivation of growth after chilling (Andre *et al.*, 2022a). It is induced by chilling in dormant buds, and *ft1* mutants fail to undergo bud break after chilling treatment (Andre *et al.*, 2022a; Sheng *et al.*, 2023). Thus, *FT1* clearly is important for reactivating growth after chilling, but its specific function in dormancy release and/or bud break is not well understood (Nilsson, 2022). One speculation is that *FT1* may be involved in the regulation of dormancy sphincters, as the up-regulation of *FT1* coincides with the removal of PD callose plugs (Rinne *et al.*, 2011), However, studies in *ft1* mutant poplar plants have shown that the removal of dormancy sphincters after chilling treatment is not affected, suggesting that *FT1* may not be necessary for PD regulation and that its function may be linked with competence to undergo bud break (Andre *et al.*, 2022a). The movement of FT protein is crucial for sustaining growth in long days and therefore one possibility is that local movement of FT1 within the shoot apex could play a role in dormancy release, as PD trafficking is involved in intercellular movement of FT in many plants. However, *FT1* transcript is broadly observed in the shoot apex, embryonic leaves, and vasculature, which makes it unclear whether FT1 movement is necessary for dormancy release (Andre *et al.*, 2022a). However, transcript levels may not necessarily correlate with protein level. Furthermore, *in situ* transcript localization is not quantitative, and thus transcripts may not be uniformly expressed across its expression domain. Finally, expression analysis of *FT1* was performed at one specific time point and therefore the localization dynamics at other times are unknown. Thus, the role of FT1 movement needs to be further addressed experimentally. Furthermore, *FT1* is specifically induced by a long period of low temperature, but the upstream regulators are still unknown. Previous studies have shown that SVL can directly bind to the *FT1* promoter to repress its induction (Tylewicz *et al.*, 2018), but this alone does not explain the hyperinduction of *FT1*. There are likely to be other regulators, particularly activators involved in the induction of *FT1* during dormancy release, and what these are needs to be ascertained to fully uncover the precise molecular mechanisms underlying *FT1* regulation and its role in dormancy release and possibly bud break.

The subfunctionalization of *FT* has also been reported in various other species and processes, such as tuberization in potato (Navarro *et al.*, 2011), vegetative growth in maize and tomato (Lifschitz *et al.*, 2006; Danilevskaya *et al.*, 2011), bulb formation in onion (Lee *et al.*, 2013), and particularly in underground overwintering bud dormancy release in the herbaceous perennial plant *Gentiana trifolia* (Takahashi *et al.*, 2022). These findings suggest that the subfunctionalization of *FT* in vegetative growth, like dormancy release, may be a common feature of perennial plants.

Furthermore, *TFL1/CEN/BFT* members also play roles in seasonal vegetative growth regulation. Poplar trees with knockdown or knockout of *CEN1/CEN2* resulted in earlier onset of flowering and earlier bud break, indicating their involvement in dormancy release (Mohamed *et al.*, 2010; Sheng *et al.*, 2023). In contrast, overexpression of *CEN1* delayed bud break in field environments (Mohamed *et al.*, 2010). The antagonistic function of *FT1* and *CEN1* in dormancy release suggests that, similar to floral transition regulation in Arabidopsis (Jaeger *et al.*, 2013), the balance between *FT1* and *CEN1* may be crucial for controlling bud break in *Populus*. *PEBP* family genes have also been widely studied in other trees, although their roles in seasonal vegetative dormancy regulation are less well reported. Recent studies in kiwifruit showed that mutation of the kiwifruit *BFT* homolog (*AcBFT2*) resulted in an evergrowing phenotype with increased branching (Herath *et al.*, 2022). *AcBFT2* is predominantly expressed in dormant axillary buds, and its evergreen loss-of-function phenotype suggests a role in axillary bud dormancy in kiwifruit (Herath *et al.*, 2022). Limited information is available on the function of *MFT*-like genes in trees, although studies conducted in herbaceous plants suggest their involvement in seed dormancy control (Xi *et al.*, 2010; Nakamura *et al.*, 2011). The *PEBP*-based mechanism for seasonal growth has also been proposed in conifers based on population genetic studies and their seasonal expression patterns (Gyllenstrand *et al.*, 2007; Karlgren *et al.*, 2013; Chen *et al.*, 2014). This suggests that an ancestral function of plant *PEBP* genes could be broader than simple regulation of flowering (Karlgren *et al.*, 2011). Advancements in transgenic technology, the application of CRISPR/Cas9 gene editing, and the availability of extensive genomic information are expected to facilitate further discoveries in the multifaced functions of tree *PEBP* family genes.

## Bud dormancy release and vernalization—similar in appearance but each goes its own way

Many studies have indeed highlighted the similarities between the vernalization process in Arabidopsis and bud dormancy release in trees (Brunner *et al.*, 2014). Similar to the seasonal dormancy in perennial trees, vernalization is an adaptive trait in many winter-annual and biennial plants that helps prevent flowering prior to winter and permits flowering in the favorable conditions of spring (Kim *et al.*, 2009). Despite both processes requiring a period of winter chilling for flowering and dormancy release, there are substantial differences (Rohde and Bhalerao, 2007; Brunner *et al.*, 2014; Rios *et al.*, 2014; Conde *et al.*, 2019). Vernalization only occurs in actively dividing cells, regardless of their location in the plant, while vegetative bud dormancy release occurs after termination of cell division (Wellensiek, 1964; Velappan *et al.*, 2017). In this respect, the ‘dormancy release’ process in *Rosaceae* flower buds is more similar to vernalization. However, chilling facilitates floral development in perennial reproductive buds, while vernalization is involved in floral initiation.

Despite the substantial differences (and a few similarities) between bud dormancy and vernalization, comparisons between them are nevertheless conceptually useful, although caution should be exercised while interpreting dormancy from a vernalization perspective. In Arabidopsis, vernalization involves an epigenetic silencing of the floral repressor *FLC*, the gene belonging to the MADS-box family (Angel *et al.*, 2011). Considering the possible commonalities between chromatin dynamics at the *FLC* locus during vernalization and the *DAM/SVP* gene locus during axillary bud dormancy, researchers have focused on studying histone modifications of individual *DAM/SVP* genes in these processes. Indeed, there has been correlative temporal association between histone modification changes of the *DAM/SVP* gene locus and bud dormancy in different tree species (Leida *et al.*, 2012;  Wu *et al.*, 2019; Vimont *et al.*, 2020; Zhu *et al.*, 2020). These studies provide valuable insights indicative of potential epigenetic mechanisms underlying dormancy; what is currently lacking is the demonstration of whether these temporal correlations are causal for dormancy release. The extensive molecular knowledge about vernalization serves as an important and useful reference for understanding the molecular mechanisms of bud dormancy. However, the emerging understanding of epigenetic regulation in tree bud dormancy [discussed in greater detail by Sato and Yamane (2024)] highlights significant differences, and points to new avenues for future research in this field.

## Perspectives

From early physiological studies to more molecular analysis of bud dormancy, significant insights into the process have been gained. However, many aspects remain to be uncovered. Amongst these are the quantitative nature of bud dormancy and bud break and its relationship with a wide variability of temperatures during the dormancy period. For example, it remains difficult to define the exact time point between dormancy maintenance and dormancy release as there are currently no visible markers to distinguish them. It can even be argued whether such a clear marker can be found given that dormancy is a quantitative trait and its release occurs gradually. Another aspect that needs to be better addressed is certain definitions such as what is dormancy. While eco- and endodormancy are commonly used terms, the underlying molecular basis is not satisfactorily resolved. In seeds, dormancy is typically defined as a failure to grow even under favorable conditions (Baskin and Baskin, 2004). This definition is also useful in the context of buds. Buds require cold exposure to be competent to undergo bud break. Thus, the ability to undergo bud break or not serves as a useful proxy for dormancy release. This also suggests that a switch occurs between the two states. However, time to bud break and/or at least 50% bud break is also used to analyze the state of dormancy. While this type of analysis is useful, how does one reconcile this with competence, since competence indicates only two states? Also, is a delay of bud break necessarily a consequence of dormancy release? The ability or competence of a bud to grow can be a separate process from how fast a bud can grow. Genetically, these two traits (i.e. dormancy release and bud break) can be separated; for example, mutants with significant differences in bud break timing have normal dormancy release response (i.e. they still require cold to undergo bud break) (Yordanov *et al.*, 2014; Azeez *et al.*, 2021). Thus, time to bud break may not always be a good proxy for dormancy release. It may be worthwhile reconsidering the use of time of bud break as a marker for dormancy or dormancy depth. Yet, what would be a better measure of dormancy release? Another important issue that needs to be addressed is the importance of apical versus axillary buds in the seasonal bud dormancy context. While both do show an impact of cold treatment, there are important regulatory differences. For example, the impact of day length on apical bud set can be uncoupled from that of axillary buds (Maurya *et al.*, 2020) and, in birch, axillary and apical buds show distinct responses to cold (Junttila and Hanninen, 2012). Thus, caution must be exercised when applying findings from axillary buds to apical dormancy, and vice versa. In summary, considerable progress has been made in understanding many features of bud dormancy especially in model species, but there is an urgent need now to extend these to non-model species and bridge the gap between laboratory research and whole-plant ecophysiology.
